# The Edge Detectors Suitable for Retinal OCT Image Segmentation

**DOI:** 10.1155/2017/3978410

**Published:** 2017-08-17

**Authors:** Su Luo, Jing Yang, Qian Gao, Sheng Zhou, Chang'an A. Zhan

**Affiliations:** ^1^Guangdong Provincial Key Laboratory of Medical Image Processing, School of Biomedical Engineering, Southern Medical University, Guangzhou 510515, China; ^2^State Key Laboratory of Ophthalmology, Zhongshan Ophthalmic Center, Sun Yat-sen University, 54S Xianlie Road, Guangzhou 510060, China

## Abstract

Retinal layer thickness measurement offers important information for reliable diagnosis of retinal diseases and for the evaluation of disease development and medical treatment responses. This task critically depends on the accurate edge detection of the retinal layers in OCT images. Here, we intended to search for the most suitable edge detectors for the retinal OCT image segmentation task. The three most promising edge detection algorithms were identified in the related literature: Canny edge detector, the two-pass method, and the EdgeFlow technique. The quantitative evaluation results show that the two-pass method outperforms consistently the Canny detector and the EdgeFlow technique in delineating the retinal layer boundaries in the OCT images. In addition, the mean localization deviation metrics show that the two-pass method caused the smallest edge shifting problem. These findings suggest that the two-pass method is the best among the three algorithms for detecting retinal layer boundaries. The overall better performance of Canny and two-pass methods over EdgeFlow technique implies that the OCT images contain more intensity gradient information than texture changes along the retinal layer boundaries. The results will guide our future efforts in the quantitative analysis of retinal OCT images for the effective use of OCT technologies in the field of ophthalmology.

## 1. Background

Optical coherence tomography (OCT) is the optical equivalent of ultrasonography, with the capability of capturing the depth-resolved cross-sectional images of biological tissues in vivo at near-histologic resolution [1]. Due to its noninvasiveness and high resolution, in combination with the characteristics of the eye and retinal anatomy, OCT has a rapid development of clinical applications in ophthalmology in recent years.

Quantitative analysis of retinal OCT image has been critical for reliable and efficient diagnosis of diseases such as glaucoma, age-related macular degeneration, and macular edema caused by diabetic retinopathy and for the evaluation of development of diseases, medical treatment responses, drug effectiveness, visual functions, and so forth [2–4]. Among others [5–7], automatic and semiautomated measurement of retinal layer thickness is considered as a class of key quantitative analysis. Numerous research efforts have been devoted to this topic [8–11], and these efforts have significantly promoted the clinical understanding of ocular diseases and improved the OCT technologies and their applications.

Retinal layer thickness measurement relies on accurate OCT image segmentation. For many automated segmentation algorithms, edge detection is an essential foundation [12–19], notwithstanding some methods resort to other features of the images [18]. Literature shows that diverse types of edge detection algorithms can be employed as a key step in image segmentation.  Table 1 summarizes the commonly used algorithms for retinal OCT image segmentation, including the Canny edge detector [12, 13], two-pass edge detection method [14, 15], local mean gradient-based edge tracking [16], peak detection method, Gaussian smoothing in combination with the Sobel kernel method [17, 18], and EdgeFlow technique [19]. Based on the nature of the information used in their algorithms, we can classify these different edge detection techniques into two categories, namely, the intensity-based and texture-based methods. The former category utilizes the intensity gradient in the images, whereas the latter tracks the texture changes rather than the intensity gradient.

Given the importance of edge detection for retinal image segmentation and the diversity of edge detectors, a natural question is which method gives the best edge detection outcome for current application. To our best knowledge, there is no study so far systematically evaluating the performance of the edge detectors applied for OCT image analysis. General performance evaluation of edge detection has long been interesting many researchers, but prior studies have not reached a unanimous conclusion yet because of the complexity of the problem. According to Heath et al., the challenge lies in that the edge detection performance depends not only on the algorithms themselves but also on the images applied to, the parameters used in the specific case, and the evaluation metrics [20].

Our goal in this paper is to evaluate what type of edge detectors best suit for retinal OCT image segmentation with given equivalent parameters, when measured using performance metrics that are meaningful for retinal layer thickness quantification. To this end, we first collect the commonly used edge detection methods in the literature on OCT image analysis and choose the most representative ones for comparison. We then research the edge detection performance evaluation literature to select the most relevant performance metrics that are meaningful for OCT image segmentation and adapt them when necessary. Using these metrics, we examine which of the selected edge detectors gives the best edge detection outcome when they are applied to the OCT images that we have collected from healthy subjects.

The remaining parts of the article are organized as follows. In the next section, we describe our research materials and methods, including retinal OCT image data collection, screening edge detectors for comparison by reviewing and analyzing the edge detection techniques used in prior studies on the retinal OCT image segmentation, and determining the most relevant performance evaluation metrics. In the third section, we present the comparison of the representative advanced approaches to retinal OCT image edge detection against different performance evaluation metrics. Finally, we discuss the findings and research opportunities.

## 2. Materials and Methods

We conducted this study in accordance with the Tenets of the World Medical Association's Declaration of Helsinki [21]. Ethical approvals were obtained from the Ethical Review Board of Southern Medical University, the Ethical Review Board of Sun Yat-sen University, and the Research Ethical Committee of Zhongshan Ophthalmic Center. After an introduction about the purpose of the study and explanation of the process and risks, the voluntary participants signed the informed consent for this data collection.

### 2.1. Image Data Choice and Data Collection

In this article, our goal is to evaluate the performance of edge detection algorithms for retinal OCT image segmentation. Because of their image dependence [20], edge detectors perform well for other types of images but may not give as good results when applied to retinal OCT image. Therefore, all interested algorithms are tested on retinal OCT image in this study.

**Figure 1:**
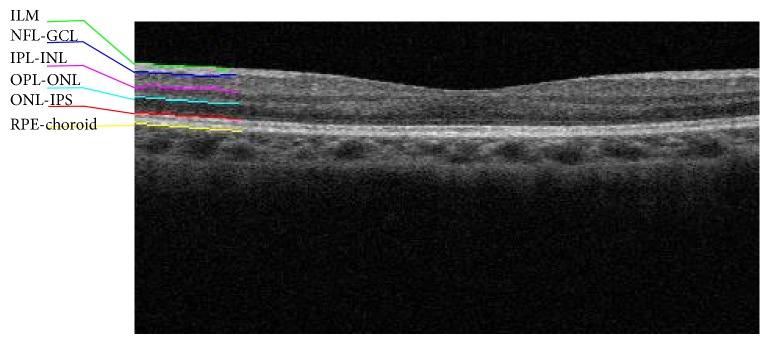
The retinal layers in a representative cross-sectional SD-OCT image. The region from the top layer ILM to the bottom layer RPE is of interest in this study and most clinical applications. Six layer boundaries are marked: ILM = the inner limiting membrane; NFL-GCL = the boundary between retinal nerve fiber layer and ganglion cell layer; IPL-INL = the boundary between inner plexiform layer and inner nuclear layer; OPL-ONL = the boundary between outer plexiform layer and outer nuclear layer; ONL-IPS = the boundary between outer nuclear layer and inner photoreceptor segment; RPE-choroid = the boundary between retinal pigment epithelium and choroid.

We collected the image data from 11 healthy volunteers (age ranges from 21 to 29, 7 males) using Topcon 3D OCT-2000 (Topcon Corporation, Tokyo, Japan) at Zhongshan Ophthalmic Center, a tertiary specialized hospital affiliated to Sun Yat-sen University, Guangzhou, China. Using 7 line 6.0 scanning mode to scan the macular area with a resolution of 1024 A-scans, we obtained the raw retinal OCT images. Due to the limitation of the software (software version: 8.20.003.04, Topcon Corporation, Tokyo, Japan), the raw images saved into bmp files have a size of 759 × 550 pixels. As the best available from our OCT equipment, this resolution is above average within those of OCT images reported in the most recent literature [3, 6, 7, 17–19].  Figure 1 shows a typical OCT image in our dataset. In this image, the 6 layer boundaries, namely, ILM, NFL-GCL, IPL-INL, OPL-ONL, ONL-IPS, and RPE-choroid are readily observable.

### 2.2. Edge Detection Algorithms

Three criteria for our choice of edge detectors for evaluation were

1. to include the algorithms that have been most commonly used in the OCT image segmentation literature,
2. to give preference to the ones representing the state of the art in edge detection, which were usually used to detect more than 3 retinal layer boundaries,
3. to include a diverse mix of algorithms utilizing different image feature information.

Using these criteria, three algorithms reviewed in Table 1 were chosen for further analysis. They are the Canny edge detector [21], two-pass algorithm [14, 15], and EdgeFlow method [22]. The former two are based on the image intensity gradient, whereas the last one is based on the image texture changes. We refer to the first two as the intensity-based edge detection methods and the third as the texture-based edge detection method. The principles of the three algorithms will be outlined below. The rest three edge detection methods, all intensity-based, were excluded from further analysis for different reasons. Among them, the edge-tracking algorithm based on the maximization of the local mean gradient was used only to detect ILM [16]; the peak detection method [17] and the Sobel kernel in combination of Gaussian smoothing [18] only detect the easily detectable boundaries ILM and OS/RPE.

#### 2.2.1. Canny Edge Detector

The Canny edge detection algorithm [23] is now generally regarded as the “standard” for edge detection in the field of digital image processing ([24], Chapter 10). The Canny edge detector works in a multistep process to detect a wide range of edges in images. At first, the image is smoothed using a linear Gaussian filter. Then, a 2D first derivative operator is utilized on the smoothed image to compute the derivatives in both the vertical and the horizontal orientations. The gradient magnitude is calculated as the root sum of squares of the derivatives in two orthogonal directions and the gradient phase as the arctangent of their ratio. Candidate edge pixels are identified as the pixels that survive after a thinning process called nonmaximal suppression. In this process, the edge strength of each candidate edge pixel is set to zero if its gradient magnitude is not larger than the gradient magnitude of the two adjacent pixels in the gradient direction, and the pixel whose gradient magnitude is the local maximum is preserved. At last, hysteresis thresholding is used to eliminate weak edge points and track the possible edge pixels. In this step, double-threshold T1 and T2 with T2 > T1 are applied; all candidate edge pixels below the lower threshold T1 are set to zero, and all pixels above the lower threshold T1 can be connected to any pixels above the higher threshold T2 through a chain of edge pixels which are labeled as edge pixels. The hysteresis helps in ensuring that the noisy edges are not broken into multiple edge fragments. In the Canny edge detection algorithm, three parameters are incorporated, which play a decisive role for detecting the result. One is the width of Gaussian filter (i.e., standard deviation of the Gaussian, *σ*). An increase in the width of Gaussian filter reduces the detector's sensitivity to noise, but blurs the image and results in loss of finer edge details. The other two are the lower threshold (T1) and the higher threshold (T2), respectively. The higher threshold should be set reasonably high and the lower threshold quite low for good detection results, because if it is too high, the lower threshold causes edge fragments and if too low, the higher threshold increases false alarms and undesirable edge fragments in the edge detection output.

#### 2.2.2. Two-Pass Edge Detection Algorithm

Two-pass edge detection algorithm is designed exclusively for detection of retinal layer in OCT images by Bagci et al. [14, 15]. The feature of edges in retinal OCT image, extending along the horizontal direction with a gentle up and downslope, was taken into account in the algorithm. The edge detection kernel *L*(*x*, *y*) is based on the first derivative of Gaussian in the vertical direction:

(1) $$\begin{array}{c} L\left(x,y\right)=-p\frac{x}{{\pi \sigma }^{2}}e^{-\left(\left(x^{2}+y^{2}\right)/2\sigma^{2}\right)}. \end{array}$$

The parameter *p* determines the polarity of edges and takes values either 1 or −1. The edge detection kernel is applied twice with alternating values of *p*. On the first pass, the boundaries between each pair of adjacent bright and dark regions, with bright on the top, such as NFL-GCL, IPL-INL, and OPL-ONL, are extracted with *p* = 1. On the second pass, boundaries between each pair of adjacent bright and dark regions, with dark on the anterior, such as ILM, INL-OPL, and ONL-IPS, were detected with *p* = −1. The peak values are marked as edges, using nonmaximal suppression and hysteresis thresholding. Satisfactory results can be obtained by adjusting the value of *σ*.

#### 2.2.3. EdgeFlow Technique

The EdgeFlow technique is a novel boundary detection scheme proposed by Ma and Manjunath [22]. The technique for boundary detection based on EdgeFlow utilizes a predictive coding model to characterize the direction of change in color (intensity of grey image) and texture at each image location at a given scale and constructs an EdgeFlow vector. By propagating the EdgeFlow vectors, the boundaries can be detected at image locations which encounter two opposite directions of flow in the stable state. Differing from intensity-based detection methods that focus on finding the local gradient maximum, EdgeFlow technique computes the directions of edge energy according to intensity or texture in an image and associated probabilities. The edge energy and corresponding probabilities obtained from different image attributes are pooled together to form a single edge field for boundary detection:

(2) $$\begin{array}{c} E\left(s,\theta \right)=\sum_{a\in A}E_{a}\left(s,\theta \right)\cdot \omega \left(a\right),\;\sum_{a\in A}\omega \left(a\right)=1,\\ P\left(s,\theta \right)=\sum_{a\in A}P_{a}\left(s,\theta \right)\cdot \omega \left(a\right), \end{array}$$

where *E*_*a*_(*s*, θ) and *P*_*a*_(*s*, θ) represent the energy and probability of the EdgeFlow computed from image attribute *a*, *a* ∈ {intensity/color, texture}; *ω*(*a*) is the weighting coefficient associated with image attribute *a*. The edge flow direction is estimated as follows:

(3) $$\begin{array}{c} \Theta \left(s\right)=\underset{\theta }{\arg\;\max}\left\{\sum_{\theta \leq \theta^{\prime }<\theta +\pi }P\left(s,\theta^{\prime }\right)\right\}. \end{array}$$

The EdgeFlow vector is then defined as

(4) $$\begin{array}{c} \vec{F}\left(s\right)=\sum_{\Theta \left(s\right)\leq \theta <\Theta \left(s\right)+\pi }E\left(s,\theta \right)\cdot \text{exp}\left(j\theta \right), \end{array}$$

where $\vec{F\left(s\right)}$ is a complex number with its magnitude representing the resulting edge energy and phase representing the flow direction. After the EdgeFlow vector of an image is computed, boundary detection can be performed by propagating the EdgeFlow vector and identifying the locations where two opposite flow directions encounter each other. The scheme facilitates integration of intensity and texture into a single framework for boundary detection.

### 2.3. Performance Evaluation Using Ground Truth

According to Heath et al. [20], edge detection performance evaluation can be classified into theoretical and empirical approaches. The former uses pure mathematical analysis without the algorithms ever being applied to an image. It has major limitation for not being able to deal with the complexity of modern edge detection algorithms. The latter can be further classified into (1) evaluation using ground truth and (2) evaluation without ground truth. Our goal in this study is to examine how different edge detectors give the best results for OCT retinal layer segmentation. Ultimately, we hope to identify the most reliable and efficient edge detectors to help doctors automate the measurement of retinal layer thickness in order to make quantitatively informed medical decisions. Our human vision systems are the most complex and efficient machine for image analysis, including edge detection. Therefore, for our intended application, the most appropriate evaluation approach should be evaluation using ground truth, which measures the difference between the algorithm-detected edges and the human-detected edges.

Due to the importance of edge detector performance evaluation using ground truth, researchers have developed numerous metrics. These metrics can be largely classified into three categories. The first category, which we refer to as the edge presence accuracy metrics (EPAM), focuses on to which extent the detected edges coincide with the ground truth without considering location shift. EPAM include mainly four metrics [25–28], namely, true positive rate, false positive rate, false negative rate, and total edge detection accuracy. The first three, respectively, measure the ratio of true edge pixels, falsely detected edge pixels, and missed edge pixels to the number of total edge pixels in the ground truth, and the fourth is the ratio between the total true edge pixels and true nonedge pixels and the total number of pixels in the region of interest. The second category, which we refer to as the edge location accuracy metrics (ELAM), focuses on the extent of edge shifts [29] introduced by the edge detection algorithms as compared to the ground truth. Metrics in this category include Hausdorff's distance [30], which measures the similarity between two images, and mean localization deviation (MLD). The third category takes into account both location accuracy and edge presence. Typical metrics include figure of merit (FOM) [31] and its expanded version (expanded FOM) [32] and multifeature quality measurement [25].

#### 2.3.1. Criteria for Performance Evaluation Metrics

OCT retinal layer segmentation aims to automate retinal layer thickness measurement in order to free ophthalmologists from laborious manual tracing of the layer boundaries. The ideal layer edge detector would give the same thickness measures to those from ground truth specified by human observers. However, even experts could not arrive at the same segmentation for a given retinal OCT image [33]. This is because manual segmentation is subject to human subjectiveness. The ground truth used for the evaluation is not really the ultimate truth. Thus, it is important to note that the traditional edge presence accuracy metrics, the probabilities of true positive, false positive (spurious edges), and missing edge, cannot offer the complete evaluation of edge detector performance. We propose that the performance metrics need to meet the criteria as follows:

1. To measure the edge presence accuracy by calculating the rates of true positive, false positive, true negative, and false negative (missing)
2. To measure edge location accuracy by calculating the signed and unsigned edge shift distance
3. To allow edge shift when calculating edge presence accuracy
4. To examine the computational costs.

#### 2.3.2. Evaluation Metrics for this Study

Based on our analysis of existing performance metrics in the literature and the metric criteria discussed previously, we choose the figure of merit (FOM, Pratt) [31], true positive rate (TPR), false positive rate (FPR), accuracy (ACC), and mean localization deviation (MLD) [34] as the basis to develop our procedure for comprehensive evaluation of the chosen edge detectors. In the paragraphs to follow, we outline the principles of these metrics.

*(1) Pratt's Figure of Merit*. FOM [31] is a classical metric utilized by numerous researchers for evaluating the performance of edge detection algorithms [25, 26, 32, 35, 36]. The definition of the FOM is given by

(5) $$\begin{array}{c} FOM=\frac{1}{\text{max}\left(N_{I},N_{A}\right)}\overset{N_{A}}{\underset{i=1}{\sum }}\frac{1}{1+\alpha d^{2}\left(i\right)}, \end{array}$$

where *N*_I_ and *N*_A_ represent the number of ideal and actual detected edge pixels, *d*(*i*) denotes the distance between the *i*th detected edge pixel and its correct position, and *α* is the scaling constant (normally set at 1/9) that is applied to provide a relative penalty between smeared edges and isolated, but offset, edges.

*(2) Edge Presence Accuracy*. The criteria on which the FOM of Pratt is based include missed valid edges, localization errors, and false alarms. Different configurations of detected edges may yield equal FOM value [35]. In order to decompose the sources of difference, Yin et al. [27] developed three metrics (TPR, FPR, and ACC) that are defined as follows.

True positive rate (TPR):

(6) $$\begin{array}{c} TPR=\frac{TP}{TP+FN}=\frac{TP}{N_{I}}. \end{array}$$

False positive rate (FPR):

(7) $$\begin{array}{c} FPR=\frac{FP}{FP+TN}=\frac{FP}{N-N_{I}}. \end{array}$$

Accuracy (ACC):

(8) $$\begin{array}{c} ACC=\frac{TP+TN}{TP+FP+TN+FN}=\frac{TP+TN}{N}. \end{array}$$

In these equations, TP (true positive) and TN (true negative) represent the numbers of correctly detected edge pixels and nonedge pixels. FP (false positive) is the number of pixels not belonging to edge but recognized as one by the algorithm, and FN (false negative) is the number of pixels belonging to edge but failed to be recognized by the algorithm. *N* is the total number of pixels within the ROI of the image, and *N*_I_ is the number of ideal edge pixels.

Given the large number (*N−N*_I_) of nonboundary pixels in the images, FPR calculated in the form of (7) is close to zero, making the metrics insensitive to the change of edge detection algorithms. We redefined it as

(9) $$\begin{array}{c} FPR=\frac{FP}{FP+TP}=\frac{FP}{N_{A}}, \end{array}$$

where *N*_A_ denotes the number of pixels of the actually detected edges.

*(3) Edge Location Accuracy Metrics*. It is known that some image processing procedures cause the shift of detected edges ([29], Chapter 3, p. 56). In order to characterize the extent to which the results from edge detection algorithms deviate from the ground truth, we introduce the location accuracy metrics, the mean localization deviation (MLD) in the context of OCT image analysis:

(10) $$\begin{array}{c} MLD=\frac{1}{N_{I}}\sum_{N_{I}}\left(\frac{1}{N_{b}}\sum_{N_{b}}d\left(i\right)\right), \end{array}$$

where *N*_*b*_ is the number of edge pixels in searching neighborhood of a ground truth edge pixel, *N*_I_ the number of pixels in the ideal edge, and *d*(*i*) the Euclidean distance of the current edge pixel in ground truth and edge pixels in searching neighborhood. For the retinal OCT images, we limited the searching neighborhood to be within 3 pixels of the true edge along each A-scan.

*(4) Adjusted TPR, FPR, and ACC*. Some procedures of image processing can introduce edge shift ([29], Chapter 3, p. 56–74). As a result, the detected edge may not match the position of actual edge. As the goal of retinal OCT image segmentation is to extract the contours of retinal layer boundaries and measure the thicknesses of different retinal layers, small and constant shifts do not have effective impact when the layer thicknesses are of the only interest. Therefore, edge pixels in the neighborhood detected by the algorithms may be accepted into true positive edge pixels when calculating the edge presence metrics. In this case, FOM, the true positive rate, and false positive rate and accuracy measures need also to be adjusted. We define these adjusted metrics as FOM_ADJ_, TPR_ADJ_, FPR_ADJ_, and ACC_ADJ_:

(11) $$\begin{array}{c} {FOM}_{ADJ}=\frac{1}{\text{max}\left(N_{I},N_{A}\right)}\overset{N_{A}}{\underset{i=1}{\sum }}\frac{1}{1+\alpha {d_{ADJ}}^{2}\left(i\right)},\\ {TPR}_{ADJ}=\frac{{TP}_{ADJ}}{N_{I}},\\ {FPR}_{ADJ}=\frac{{FP}_{ADJ}}{N_{I}},\\ {ACC}_{ADJ}=\frac{{TP}_{ADJ}+TN_{ADJ}}{N}, \end{array}$$

where *N*_I_ and *N*_A_ represent the number of ideal and actual detected edge pixels, *d*_ADJ_(*i*) is the distance between the *i*th detected edge pixel and its correct position, TP_ADJ_ is the number of edge pixels detected by an algorithm that are considered as edges within the neighborhood of the ground truth, FP_ADJ_ is the number of false positive pixels after the neighborhood searching and edge pixel adjustment, and TN_ADJ_ equals TN as it is not affected by the neighborhood adjustment. These adjusted metrics allowing the edge shift can better reflect the amount of detected edge points.

#### 2.3.3. Evaluation Procedure

**Figure 2:**
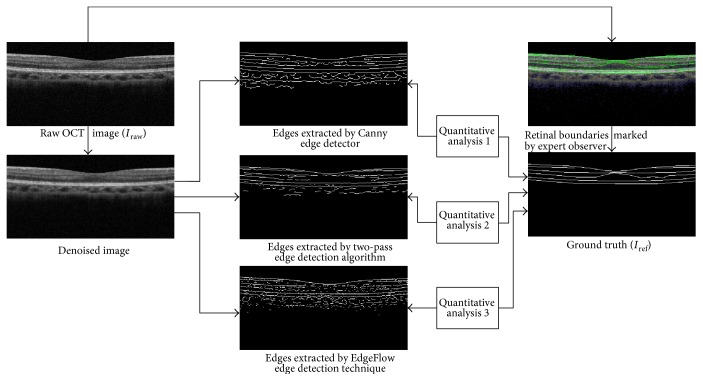
Procedures of performance evaluation of edge detectors. The ground truth of OCT retinal layer boundaries is labeled by an expert observer. Raw OCT images are firstly denoised and then applied with three automatic edge detection algorithms (i.e., Canny, two-pass, and EdgeFlow) to obtain the algorithm-detected boundaries, which are compared with the ground truth.

The major steps for performance measurement include the preparation of ground truth, the preprocessing of OCT images, the application of edge detectors with appropriate parameters to obtain the near optimal outcome for each detector, and using the performance metrics to evaluate the goodness of edge detectors against the ground truth.  Figure 2 summarizes the flow of performance evaluation.

*(1) Ground Truth Preparation*. We asked an expert observer to manually delineate the edges for representative retinal OCT images to form a base dataset of ground truth, as noted by *I*_ref_ in Figure 2. Because the ILM and RPE are the outer boundaries of the retinal structure and they are strong edges that can usually be reliably detected, we define the images between ILM and RPE (included) as the region of interest (ROI). Only those edges within the ROI are extracted for comparison with the ground truth.

**Figure 3:**
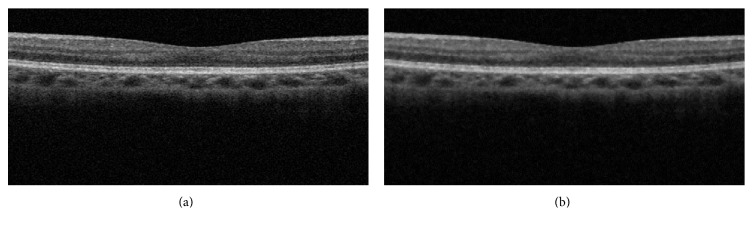
Median filtering of the retinal OCT image. (a) Raw retinal OCT image and (b) the denoised image.

*(2) Image Preprocessing*. Before applying the computer algorithm for each edge detector, we conducted necessary image preprocessing. Due to constructive or destructive interference of the light waves from the object, spectral domain retinal OCT images suffer from the inherit speckle noise [37], which decreases the quality of image and causes unreliable retinal layer segmentation. In order to improve the quality of edge detection, preprocessing becomes a necessary step. We first converted the raw OCT image bmp files into gray-scale images and cropped the images to the region of interest (ROI, 200 by 400 pixels) in this study. The literatures have suggested the use of filters like mean, median, and Gaussian [38–40] for noise removal. We choose median filtering to remove the speckle noise. The original retinal OCT image and the denoised image are shown in Figure 3().

*(3) Edge Detection*. We randomly chose 8 images from our database of raw OCT retinal images and apply the three edge detectors. As discussed earlier, the edge detection outcomes may be influenced not only by the algorithm itself but also by the input parameters [20]. We varied the parameters systematically to obtain the optimal possible edge outcomes for each of the edge detection algorithms.

*(4) Performance Evaluation*. In edge detection performance evaluation step, we compared the edge detection outcomes from the three computer algorithms against the human manually traced retinal layer boundaries. We applied the metrics that were broadly used in the literature and relevant to our specific research context and purpose. We also applied the adjusted metrics developed and discussed in the previous section. Finally, we examined the evaluation outcomes in terms of both differences and relationships.

## 3. Experiments and Results

We implemented all the data analysis in MATLAB R2012a (The Mathworks Inc., MA, USA) on a personal computer running Windows 7 operating system with an 3.60 GHz Intel® Core™ i7 CPU and 4 GB of memory. The raw image data was acquired and stored into bmp files. Data in the intermediate analysis steps were computed and stored in the double precision data format in order to minimize digitization errors. Three edge detection algorithms were carefully coded and double checked for the correctness.

### 3.1. Edge Detection

#### 3.1.1. Input Parameters

Input parameters can significantly influence the resulting edge quality for given edge detection algorithm [20]. In selecting values for these parameters, we are interested in finding the set that provides good edge detection accuracy, that is, the boundaries coinciding the six retinal layers as shown in Figure 1 with high signal-to-noise ratio. In terms of performance evaluation metrics, good parameters give the high values of FOM, TPR, ACC, and their adjusted forms and lower values of FPR and MLD. Through applying the three detection methods with multiple sets of parameters on OCT retinal images (*n* = 21), the edge detection results were obtained and compared. By observing the outcomes, we chose the parameter set for each algorithm as follows. In Canny edge detection, the best result can be obtained by setting the width of the Gaussian filter *σ* = 3, the lower threshold T1 = 0.005, and the upper threshold T2 = 0.1; for two-pass edge detection algorithm, the width of Gaussian *σ* = 3, consistent with Bagci et al. [14], the lower threshold T1 = 0.005, and the upper threshold T2 = 0.15, similar to those for Canny edge detector; for the EdgeFlow algorithm, we followed Ma and Manjunath [22] and chose the equal weighting coefficients for intensity and texture, that is, *ω*(intensity) = *ω*(texture) = 0.5.

#### 3.1.2. Edge Detection Results

**Figure 4:**
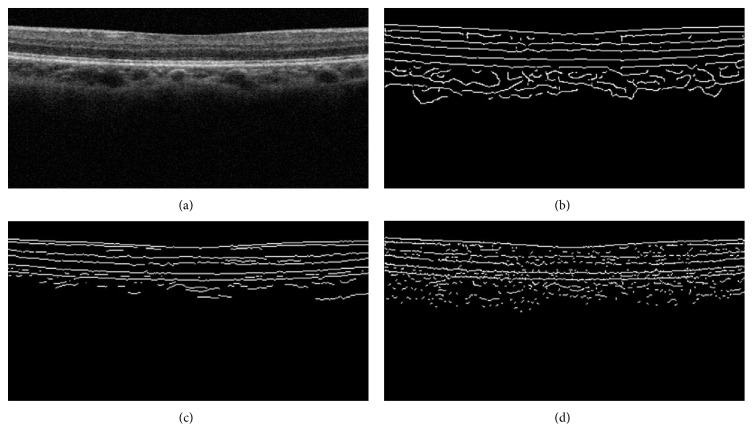
Detection results from three edge detection methods. (a) Original retinal OCT image, (b) Canny detector, (c) two-pass edge detection algorithm, and (d) EdgeFlow edge detection technique.

Figure 4(a) shows an original retinal OCT image. Figures 4(b), 4(c), and 4(d) show the edge detection outcomes from the Canny edge detector, two-pass edge detection technique, and EdgeFlow algorithm, respectively. From these edge detection results, six retinal layer boundaries of our interest are readily identifiable, although with some noises caused by false positives and boundary breakages caused by the false negatives.

**Figure 5:**
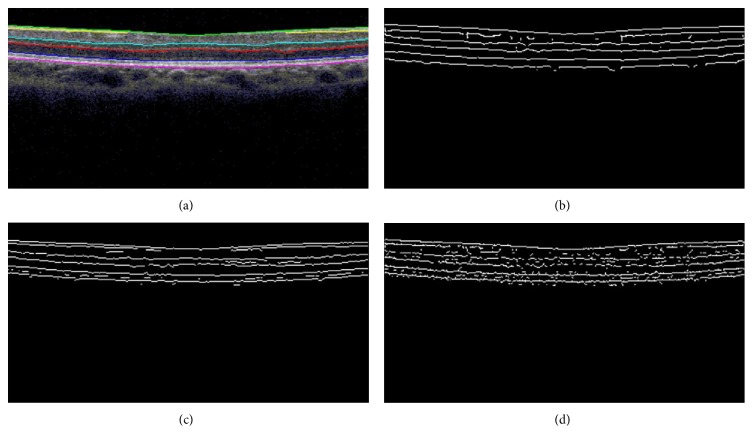
Edge extraction in ROI, from human observer and three algorithms. (a) Retinal layer boundaries marked by a human observer, (b) edges within ROI for the Canny edge detector, (c) edges within ROI for two-pass edge detection algorithm, and (d) edge within ROI for EdgeFlow technique.

For our purpose in this study, we were mainly interested in how the three edge detectors performed in detecting the 6 retinal layer boundaries, which were also the key information in the literatures for retinal layer thickness measurement [8–11]. We defined the region of interest (ROI) to be the area between the ILM and RPE that are the most outer boundaries of the retinal structure.  Figure 5(a) is the OCT retinal image with overlaid ground truth edges marked by an expert observer. Figures 5(b), 5(c), and 5(d) show the edges within ROI detected by the three algorithms, which will be the basis of performance evaluation in the next section. Visually, the result from the Canny edge detector in Figure 5(b) shows a well-defined six boundaries, although with some breakages and noises. The result from the two-pass method shown in Figure 5(c) gives more than 6 layers in some locations, but in general, the six layer boundaries of interest are very clear with less breakages compared to those in Figure 5(b). The result from EdgeFlow algorithm depicted in Figure 5(d) shows more breakages and more noises, although all 6 layer boundaries are still recognizable.

### 3.2. Performance Evaluation

**Figure 6:**
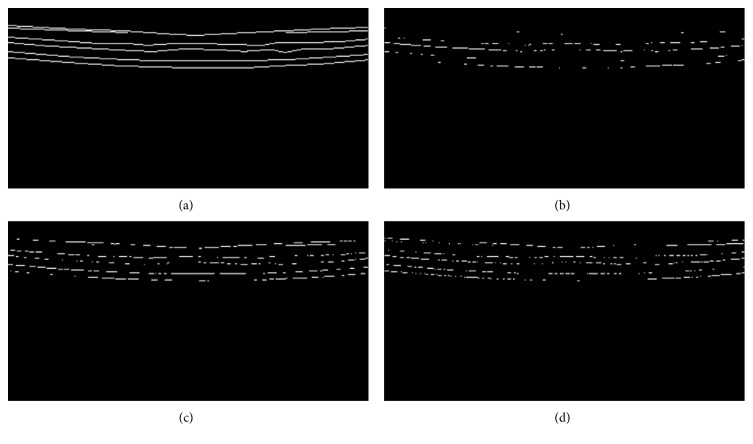
Edge pixels from three edge detectors overlapping with the ground truth. (a) The ground truth edges marked by human observer. (b) The true positive points obtained by Canny edge detector. (c) The true positive points obtained by two-pass edge detection algorithm. (d) The true positive points obtained by EdgeFlow technique.

To quantify the performance of the three edge detectors, we use three sets of measurements discussed in Materials and Methods. The first set of metrics include FOM, TPR, FPR, and ACC, which have been broadly used in the literature [20, 25–28, 30–32] when evaluating edge detectors on images other than the OCT retinal images. In order to calculate TPR, FPR, and ACC, we use the ground truth as a template to screen the coincided edge pixels from the three edge detection algorithms.  Figure 6 shows the edge points overlapping with the manually traced edges (ground truth).

The calculated performance metrics are summarized in Table 2. Based on the mean values of FOM for the three edge detectors, it seems that the best performer is the two-pass method.  Table 3 summarizes the results of the statistical analysis. Two-sample *t*-tests confirmed the impression that two-pass method significantly outperforms both the Canny edge detector (*p* = 1.19*e* − 6), and EdgeFlow (*p* = 5.28*e* − 4) in terms of FOM. In addition, the EdgeFlow algorithm outperforms the Canny edge detector (*p* = 4.65*e* − 3). The TPR for two-pass is also significantly higher than that for Canny (*p* = 4.29*e* − 4) and that for the EdgeFlow method (*p* = 0.0058). The same pattern occurs when measured with ACC. ACC for the two-pass method is significantly higher than that for the Canny (*p* = 3.15*e* − 3) and EdgeFlow method (*p* = 5.61*e* − 4). On the other hand, FPR for two-pass method is significantly lower than that for Canny (*p* = 0.00024) and EdgeFlow (*p* = 2.73*e* − 04). All the metrics suggest that two-pass method is the best among the three. However, Canny and EdgeFlow methods are not significantly different when measured using TPR (*p* = 0.2045), FPR (*p* = 0.3727), or ACC (*p* = 0.3304).

However, when comparing Figures 5(b), 5(c), and 5(d) and Figures 6(b), 6(c), and 6(d) one by one, the Canny edge detector results in much less edge pixels on locations of the ground truth, although its outcome seems good as well in Figure 5(b). This suggests that the Canny edge detector introduced edge shifts and resulted and lower performance score in Tables 2 and 3. To examine the possibility, we calculated the mean localization deviation (MLD) for the three edge detectors. The results in Table 4 indeed show the largest mean value of MLD for the Canny edge detector. Two-sample *t*-tests (Table 5) confirmed that the MLD value for the Canny edge detector is significantly higher than that for the two-pass edge detection algorithm (*p* = 0.04) and almost significantly higher than that for the EdgeFlow method (*p* = 0.058).

**Figure 7:**
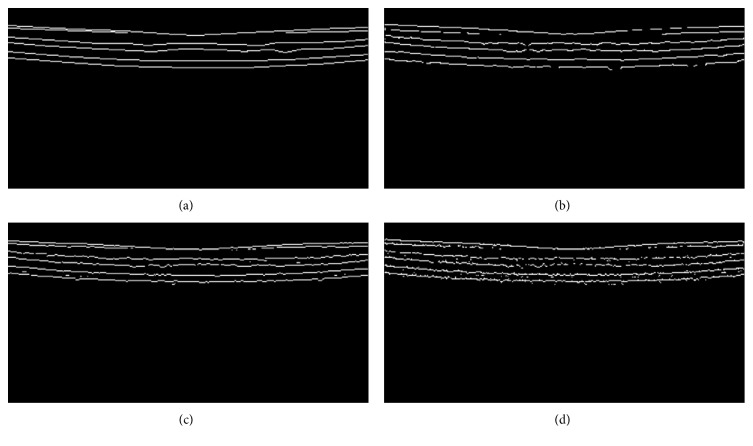
Edges for the adjusted performance metrics. (a) The ground truth edges, (b) the edge pixels for Canny edge detector, (c) the edge pixels for the two-pass edge detection algorithm, and (d) the edge pixels for the EdgeFlow technique.

For OCT retinal image layer thickness measurement, if the edge shift is within a small range and to the same direction, the outcome may not be significantly influenced. Therefore, we examine how the edge detectors perform when measured with a second set of metrics; the adjusted measures include FOM_ADJ_, TPR_ADJ_, FPR_ADJ_, and ACC_ADJ_, which were developed in Materials and Methods.  Figure 7(a) is the ground truth edges; Figures 7(b), 7(c), and 7(d), respectively, show detected edge pixels within 2 pixels searching neighborhood corresponding to its ground truth edge for the three edge detectors. Visually, the outcome for Canny edge detector is much improved when compared to that in Figure 6(b); the edges for EdgeFlow method are much noisy. Quantitative metrics for the edge detectors based on Figure 7 are summarized in Table 6.

The results for FOM, TPR, ACC, and FPR after the adjustment (Table 6) are all better than those before the adjustment (Table 2). This finding is reasonable in that the false alarm reduces, and FOM, TPR, and ACC increase, when more detected pixels are considered as correct edge. Note that in both tables, the higher values for FOM, TPR, and ACC mean the better performance, whereas for FPR, the lower the value, the better the performance.

Detailed statistical analysis on the performance of three edge detectors measured with the adjusted metrics is summarized in Table 5. For FOM_ADJ_ measure, the two-pass is again significantly better than both Canny edge detector (*p* = 4.41*e* − 11) and the EdgeFlow method (*p* = 2.61*e* − 6); EdgeFlow is also significantly better than Canny (*p* = 8.13*e* − 7). When measured with TPR_ADJ_, FPR_ADJ_, and ACC_ADJ_, Canny does not differ significantly from the two-pass method (*p* = 0.3933, *p* = 0.4613, and *p* = 0.4175, resp.); a similar pattern (except for TPR_ADJ_) occurs for Canny and the EdgeFlow method (*p* = 0.01945, *p* = 0.1471, and *p* = 0.1635, resp.). However, these measures show that the two-pass method is significantly better than the EdgeFlow technique (*p* = 8.1322*e* − 05, *p* = 2.41*e* − 04, and *p* = 3.2125*e* − 05, resp.).

Finally, we noticed in our experiments that the EdgeFlow technique took much longer time for each edge detection task. When the time was measured for processing a sample of OCT retinal images (200 by 400 pixels in the ROI), the average computational time is 2.77 ± 1.24 seconds, 3.85 ± 0.18 seconds, and 467.66 ± 1.33 seconds, respectively, for two-pass, Canny, and EdgeFlow methods, confirming that the EdgeFlow approach took a significantly larger amount of time than the other two algorithms.

## 4. Discussion and Conclusions

In this study, we intended to search for the edge detectors that best suit for the OCT retinal image segmentation task. With the analysis of literature and our experiment, we have identified the most promising candidate algorithms, namely, Canny edge detector, the two-pass method, and the EdgeFlow technique. Using the performance evaluation metrics (FOM, TPR, FPR, and ACC) and their adjusted versions (FOM_ADJ_, TPR_ADJ_, FPR_ADJ_, and ACC_ADJ_), we examined the three methods applied to the realistic OCT retinal images. Our results show that the two-pass method consistently outperforms the other two. In addition, the MLD metrics shows that the two-pass method caused smaller edge shifting problem. Although the computational cost for the two-pass method is slightly higher than the Canny edge detector, it is over 100 times lower than that for the EdgeFlow technique. Based on the above analysis and findings, we conclude that the two-pass method is among the three the best approach to edge detection for the OCT retinal layer image segmentation task. Furthermore, the outperformance of two-pass method measured by the original and adjusted metrics and the advantage of Canny edge detector over EdgeFlow technique in terms of FOM_ADJ_ and TPR_ADJ_ and MLD lead to another conclusion that the intensity-based edge detectors outperform the texture-based edge detector for OCT retinal image analysis.

The findings in the study suggest that it is critical to use the most appropriate algorithms to detect the retinal layer boundaries in the OCT images in order to automate the quantitative analysis of retinal OCT images. Combined with the findings in the literature that EdgeFlow method significantly outperformed Canny algorithm in texture segregation tasks [22], this study offers support to the idea that the performance of edge detectors is image property dependent [20] as both Canny and two-pass methods surpass EdgeFlow in the current application. In line with this thought and findings, it is necessary argue that the best performer for normal retinal OCT images also work best for pathological retinal images. Additionally, the intensity-gradient based methods (two-pass and Canny algorithms) outperforming texture-based method (EdgeFlow) might suggest that the OCT images contain more intensity gradient changes than texture changes along the longitudinal direction. The relative weight of intensity and texture information in OCT retinal image warrants further study in the future.

With the development of OCT technologies and their applications in the field of ophthalmology, more and more data is readily available. Extracting meaningful information from the ever-increasing volume of clinical data reliably and efficiently forms the basis for modern medical decision making and research. Reliable and efficient OCT retinal image segmentation will contribute to the development of this trend. Future research efforts would need to overcome several limitations in this study. First, the input parameters used in our experiments were selected over a relatively small sample space and the decisions on the “optimal” parameters were subject to human subjectiveness. Although it is almost impossible to identify the absolutely optimal input parameters for each edge detector [20], the choice of optimal input parameters may be improved by conducting a large number of experiments and averaging opinions from more expert viewers. The second limitation in our study is the use of a single expert observer to define the ground truth. Individual subjectiveness may be reduced by averaging across multiple decisions for the ground truth. Moreover, our data were all collected from voluntary healthy subjects. If the edge detectors perform differently for different types of images, it is necessary to examine how they perform on pathological retinal images in future studies.

## Figures and Tables

**Table 1:** Summary of edge detection techniques involved in retinal OCT image segmentation studies.

Principles	Edge detection algorithms	Applications in OCT image analysis	References
Intensity-based	Canny edge detector	To obtain global gradient information	Yang et al. [12]
		To extract global boundary	Shijian et al. [13]
	Two-pass edge detection algorithm	To extract global boundary	Bagci et al. [14]
			Bagci et al. [15]
	Edge-tracking algorithm	To detect ILM	Rossant et al. [16]
	Peak detection method	To detect the easily detected boundary ILM and OS/RPE	Cha and Han [17]
	Gaussian smoothing + Sobel kernel		Lang et al. [18]

Texture-based	EdgeFlow technique	To detect global boundary of retinal OCT image	Niu et al. [19]

**Table 2:** Performance evaluation using metrics FOM, TPR, FPR, and ACC.

Edge detectors	FOM	TPR	FPR	ACC
Canny edge detector	0.36 ± 0.0865	0.22 ± 0.0816	0.75 ± 0.0881	0.98 ± 0.0026
Two-pass edge detection algorithm	0.67 ± 0.0726	0.41 ± 0.0968	0.57 ± 0.1182	0.98 ± 0.0033
EdgeFlow technique	0.50 ± 0.0942	0.27 ± 0.0875	0.79 ± 0.094	0.97 ± 0.0027

**Table 3:** Two-sample *t*-test on comparison of the edge detection performance.

Samples	FOM	TPR	FPR	ACC
Canny and two-pass	H0: m(c) > m(t)	H0: m(c) > m(t)	H0: m(c) < m(t)	H0: m(c) > m(t)
	*p* = 1.19*e* − 06	*p* = 4.29*e* − 04	*p* = 0.0024	*p* = 3.15*e* − 03

Canny and EdgeFlow	H0: m(c) > m(e)	H0: m(c) = m(e)	H0: m(c) = m(e)	H0: m(c) = m(e)
	*p* = 4.65*e* − 03	*p* = 0.2045	*p* = 0.3727	*p* = 0.3304

Two-pass and EdgeFlow	H0: m(t) < m(e)	H0: m(t) < m(e)	H0: m(t) > m(e)	H0: m(t) < m(e)
	*p* = 5.2769*e* − 04	*p* = 0.0058	*p* = 2.73*e* − 04	*p* = 5.6127*e* − 04

**Table 4:** Mean localization deviation (MLD).

Canny edge detector	Two-pass edge detection	EdgeFlow technique
1.27 ± 0.3340	1.02 ± 0.2484	1.05 ± 0.1831

**Table 5:** Two-sample *t*-test on the adjusted performance metrics and MLD.

Samples	FOM_ADJ_	TPR_ADJ_	FPR_ADJ_	ACC_ADJ_	MLD
Canny and two-pass	H0: m(c) > m(t)	H0: m(c) = m(t)	H0: m(c) = m(t)	H0: m(c) = m(t)	H0: m(c) < m(t)
	*p* = 4.41*e* − 11	*p* = 0.3933	*p* = 0.4613	*p* = 0.4175	*p* = 0.04003

Canny and EdgeFlow	H0: m(c) > m(e)	H0: m(c) < m(e)	H0: m(c) = m(e)	H0: m(c) = m(e)	H0: m(c) < m(e)
	*p* = 8.13*e* − 07	*p* = 0.01945	*p* = 0.1471	*p* = 0.1635	*p* = 0.0581

Two-pass and EdgeFlow	H0: m(t) < m(e)	H0: m(t) < m(e)	H0: m(t) > m(e)	H0: m(t) < m(e)	H0: m(t) = m(e)
	*p* = 2.6132*e* − 06	*p* = 8.1322*e* − 05	*p* = 2.41*e* − 04	*p* = 3.2125*e* − 05	*p* = 0.1074

**Table 6:** FOM_ADJ_, TPR_ADJ_, FPR_ADJ_, and ACC_ADJ_ after redefining the detected edges.

Edge detectors	FOM_ADJ_	TPR_ADJ_	FPR_ADJ_	ACC_ADJ_
Canny edge detector	0.51 ± 0.026	0.88 ± 0.0841	0.12 ± 0.0848	0.99 ± 0.0024
Two-pass edge detection algorithm	0.81 ± 0.0416	0.91 ± 0.0277	0.093 ± 0.0355	0.99 ± 0.0009
Edge flow technique	0.66 ± 0.0409	0.85 ± 0.0125	0.16 ± 0.014	0.99 ± 0.0004
